# The Role of Academic Achievement in the Relationship between School Ethos and Adolescent Distress and Aggression: A Study of Ninth Grade Students in the Segregated School Landscape of Stockholm

**DOI:** 10.1007/s10964-020-01199-w

**Published:** 2020-02-05

**Authors:** Maria Granvik Saminathen, Stephanie Plenty, Bitte Modin

**Affiliations:** 1 Centre for Health Equity Studies (CHESS), Department of Public Health Sciences, Stockholm University, Stockholm, Sweden; 2 Institute for Future Studies (IFFS), Stockholm, Sweden

**Keywords:** School ethos, School performance, Psychological well-being, School segregation, Mediation

## Abstract

Equitable access to high-quality schools is important for student achievement. However, the increasing attention placed on adolescent mental health promotion suggests that school contextual factors and school achievement may also play an important role for students’ psychological well-being. This study examined the relationships between school ethos, academic achievement, psychological distress and aggressive behaviour among Swedish students, further considering the role of school sociodemographic composition. Analyses were based on two separate data collections in Stockholm, one among teachers (*n* = 2089) and the other among students aged 15–16 (*n* = 9776; 49.7% girls). Using multilevel structural equation modelling, the relations between teachers’ reports of school ethos and students’ reports of achievement, psychological distress and aggressive behaviour were tested. Analyses showed a positive relationship between a school’s ethos and average academic achievement. At the school level, higher academic achievement was in turn associated with less psychological distress among students, providing an indirect pathway between school ethos and psychological distress. At the individual level, students with higher academic achievement reported less psychological distress and aggressive behaviour. These findings indicate that schools’ value-based policies and practices can play a role for students’ academic performance, and through this, for their psychological well-being.

## Introduction

School ethos and related notions such as school climate are some school contextual features that are recognised as central to adolescent achievement and well-being (Kutsyuruba et al. 2015). In the context of school sociodemographic segregation, such modifiable aspects of school quality have the potential to boost a school’s effectiveness, regardless of students’ socioeconomic and ethnic background (Liu et al. 2015). Most studies on school effectiveness focus on improving students’ academic performance. Although schools’ primary objectives are academic in nature, schools also embody central social arenas that are of relevance for young people’s psychological welfare (SNAE 2014). Accordingly, attending a school that offers a positive social atmosphere conducive for learning may also protect against poor psychological well-being among adolescents (Aldridge and McChesney 2018). Considering the rise in self-reported psychological health complaints among Swedish adolescents observed in the past decades (National Board of Health and Welfare 2014), it is critical to identify shared contextual features that can promote the well-being of young people. This study examines the association between school ethos in terms of teacher-rated beliefs, values and practices and adolescents’ level of self-reported poor well-being, exploring the mediating role of academic achievement.

### School Ethos

Since the 1970s, the “effective schools” movement has advocated that the quality of schools can have an effect on student academic achievement and behaviour beyond a student’s own family background and the school’s student composition (Rutter et al. 1979). So-called effective schools are characterised by better school performance (MacBeath and Mortimore 2001) and a lower degree of behavioural and psychological problems among students (Sellström and Bremberg 2006).

This study examines school ethos, a fundamental element of the broader theory of school effectiveness (Rutter et al. 1979). School ethos is employed as an exploratory measure that is considered to be distinct from the concept typically referred to as school climate (Modin et al. 2017). A school’s ethos is experienced at the teacher-level and encompasses the beliefs, values and norms that shape the way that teachers and students relate, interact, and behave towards each other (Modin et al. 2017). It refers to the values and principles guiding policy and practice at a school (Glover and Coleman 2005) that have been established at a higher level in the school structure represented by the school leadership (Pepper and Thomas 2002). The school’s ethos is in turn manifested in the way that teachers approach their work and how teachers and students interact and behave towards each other in their everyday social interplay (Modin et al. 2017). Accordingly, the strength of a school’s ethos is assessed through aspects such as the level of transparency of the school’s value system, teachers’ conduct towards students, as well as the implementation of policies regarding discipline and bullying (Kjellström et al. 2017).

Schools with a leadership that has managed to build a robust and favourable ethos are believed to be better equipped to compensate for individual socioeconomic background. A strong school ethos is also more likely to promote constructive student behaviours and a shared sense of belonging that fosters positive student outcomes (Warin 2017). Hence, even though some schools may have a sociodemographic advantage that facilitates the establishment of a favourable school ethos, such conditions have the potential to make a difference in all schools (Liu et al. 2015).

### School Ethos, Academic Achievement and Poor Psychological Well-Being

Gillander and Hammarström (2003) have highlighted that schools’ work environment can contribute to poor health among students, just as for the adult working population. Ethos as a school-contextual feature could thus be decisive for student outcomes beyond academic achievement.

The relationship between indicators of a school’s teaching and learning environment and student academic achievement has been widely established (Kutsyuruba et al. 2015). For instance, high teacher expectations on students (Brault et al. 2014), perceived support from teachers (Ahmed et al. 2010) as well as a safe and orderly school environment (Gaskins et al. 2012) have been linked with higher levels of student achievement. By contrast, high teacher turnover may undermine student performance at a school (Ronfeldt et al. 2013).

Studies have also identified associations between school-contextual features and adolescent psychological well-being. Most recently, a meta-analysis by Goldberg et al. (2019) concluded that initiatives that enhanced a school’s ethos and environment through a whole-school approach had small but positive effects on behavioural adjustment and internalising symptoms among children and young people. Undheim and Sund (2005) specifically identified teacher support as a strong predictor of the level of depressive symptoms among Norwegian adolescents, both cross-sectionally and in the following year. Thus, even if the influence of schools on student outcomes tends to be higher for indicators related to achievement than to well-being (Opdenakker and Van Damme 2000), the resources embedded in the school context may also help to reduce poor psychological well-being among students.

Considering the school’s primary function as an educational institution, the implications of academic achievement for adolescents’ psychological well-being needs greater attention. Although this relationship has often been shown to be reciprocal (Gustafsson et al. 2010), the capability of empirical studies to identify causality is limited. Nevertheless, some studies have found that academic underachievement can lead to future psychological health problems (Moilanen et al. 2010). A meta-analysis by Huang (2015) revealed a weak but significant correlation between low achievement and subsequent depression during childhood, a finding that may be particularly applicable to girls (McCarty et al. 2008). Likewise, a report by the Swedish Public Health Agency found that among girls with a performance at or below the average school performance, 61% reported psychosomatic symptoms, compared to 45% among girls who performed above average (PHAS 2016). The corresponding proportions for boys were 35% and 23%, respectively. In addition, a Finnish study found that schools with the lowest average academic performance presented with lower levels of student well-being (Karvonen et al. 2018).

Various processes could be underlying the pathway between school performance and the level of psychological well-being. Academic difficulties may lead adolescents to experience lower self-competence (Westling Allodi 2010) and self-esteem (Gustafsson et al. 2010), as well as higher levels of stress (Myklestad et al. 2011), which in turn undermine psychological well-being (Undheim and Sund 2005). Furthermore, an awareness of increasingly precarious employment prospects may contribute to school-related stress and thus reduced well-being, as young people today face a labour market that places higher demands on education and formal qualifications than previously (Andres and Wyn 2010; PHAS 2018a).

### Conceptualising Adolescent Psychological Well-Being

The World Health Organisation (WHO) has highlighted the deterioration in adolescent subjective health as a public health problem (Inchley et al. 2016). Psychological distress is a widely used indicator of psychological health in epidemiological studies, referring to a state of emotional suffering characterised by symptoms of depression and anxiety (Drapeau et al. 2012). However, responses to poor psychological well-being may also be turned outwards (National Board of Health and Welfare 2013). Such externalising problems are outer-directed and incite discomfort and conflict in others, and include aggressiveness and delinquency (Levesque 2011). The present study focuses on these two aspects of self-reported poor psychological well-being: *psychological distress* as a manifestation of internalising problems, and *aggressive behaviour* as an expression of externalising problems (Bremberg and Dalman 2015). Although psychological complaints of this milder kind do not necessarily indicate problems requiring treatment, they may still negatively affect an adolescent’s overall quality of life (National Board of Health and Welfare 2013).

### Gender Differences

A common tendency during adolescence is for boys to engage more in externalising responses to reduced psychological well-being, such as impulsive and/or aggressive behaviour towards others. By contrast, girls more commonly react in internalising ways, manifested as depressive and anxious feelings (Rescorla et al. 2007). It is also notable that girls tend to perform better in school than boys in Sweden (SNAE 2015), which could to some extent protect them from psychological problems. At the same time, research has indicated that boys and girls may respond differently to school failure (Drapeau et al. 2012), and that girls tend to report greater school-related stress (Currie et al. 2008; PHAS 2018b). Accordingly, school stress and the school context may be more influential for girls’ psychological well-being than for boys’ (Drapeau et al. 2012). As psychological problems often manifest differently in boys and girls, it is thus conceivable that school ethos and achievement may have different implications for psychological distress and aggressive behaviour among girls and boys.

### The Swedish Context

When studying school effectiveness, it is critical to contextualise schools within the wider social environment (Goldstein and Woodhouse 2000). In Sweden, the Education Act (SSB 2010) states that the prospects of achieving good marks and experiencing a favourable school context shall be as independent as possible from students’ family background, place of residence and attended school (SNAE 2012), in line with the effective schools movement (Rutter et al. 1979). Yet, in the past two decades, between-school differences in school quality and student achievement have increased, along with a more unbalanced sociodemographic distribution of students across schools (Östh et al. 2013), amplifying the importance of which school a child attends (SNAE 2018). For example, a previous study based on the same data used in the current study found teacher-rated school ethos to mediate some of the effects of school student body composition on academic achievement (Granvik Saminathen et al. 2018). In addition, grade 9 is the final year of the compulsory school in Sweden. This is a particularly critical period as students’ marks determine their options for upper secondary school, and adolescents tend to be well aware that obtaining an upper secondary school qualification substantially enhances their future employability and opportunities (SNAE 2015). Thus, when examining Swedish adolescents’ well-being, it is critical to take their academic achievement as well as the school’s sociodemographic profile into consideration in the analysis.

## Current Study

**Fig. 1:**
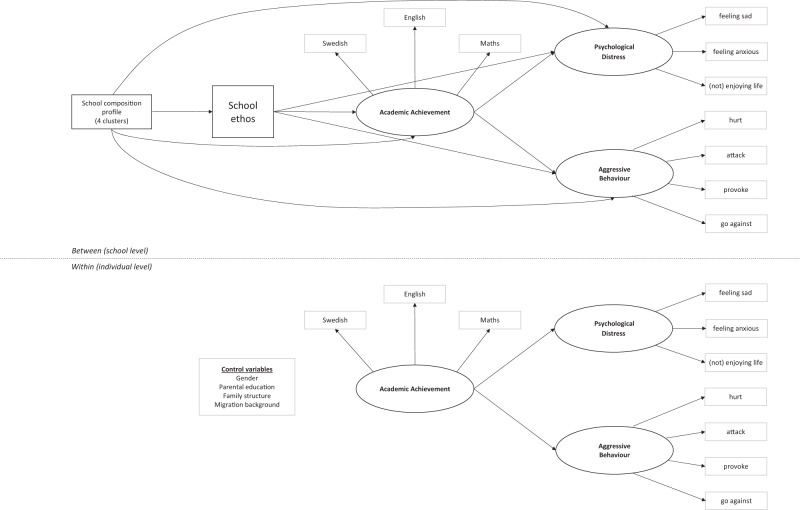
Proposed model. Correlations between the latent factors were modelled but are not shown for simplicity. The model adjusts for individual student gender, migration background, parental education, as well as family structure on each of the latent variables

This study aims to examine the role of teacher-rated school ethos for adolescent outcomes, beyond the effects of school and student sociodemographic characteristics. In line with theories of effective schools, the link between school ethos as experienced by teachers and students’ academic achievement will be studied. The effective schools literature will also be extended to adolescent well-being by conceptualising school as students’ work environment and main social context, and by exploring the role of school ethos in students’ psychological distress and aggressive behaviour. Multilevel structural equation modelling (ML-SEM) will be used to distinguish between individual versus school components of these associations. The effects of school ethos on student outcomes are examined at the school level, while the associations between academic achievement, psychological distress and aggressive behaviour are simultaneously estimated at the individual and school levels. Figure 1 illustrates the proposed model.

If positive contextual characteristics in a shared environment such a schools can promote both academic achievement and psychological well-being among students (Goldberg et al. 2019), schools with a stronger ethos are expected to have higher academic achievement, and lower psychological distress and aggressive behaviour, once the effects of gender and other sociodemographic characteristics at the student- and school-level are taken into account (Hypothesis 1). Considering that teaching and learning are the school’s primary missions and that school performance is associated with psychological well-being, a more conducive school ethos is expected to predict lower student psychological distress and aggressive behaviour, indirectly through academic achievement (Hypothesis 2). Finally, considering gender differences in the prevalence and manifestation of psychological problems, school ethos is anticipated to have a stronger positive effect on academic achievement among girls than among boys. In addition, psychological distress is expected to be more closely associated with girls’, and aggressive behaviour more strongly associated with boys’, lower academic achievement (Hypothesis 3).

## Methods

### Data

The data material is drawn from cross-sectional student and teacher surveys conducted at lower secondary schools in 2014 and 2016, as well as from the Swedish National Agency for Education’s online information system SIRIS (SNAE 2016), containing official statistics about schools. The Stockholm School Survey (SSS) is conducted every two years among ninth grade students (aged 15–16 years) in all public and most independent schools in the municipality of Sweden’s capital city Stockholm. This survey is part of the city’s prevention work with young people’s substance use and criminal behaviour. Student participation was voluntary and anonymous. Students completed the survey within 45 min during regular class time without any compensation. Parental consent was not required for the SSS because the Swedish Act concerning the Ethical Review of Research Involving Humans does not require it for minors aged 15 and older to participate in surveys.

The Stockholm Teacher Survey (STS) was sent to all teachers working in the lower secondary schools participating in the SSS. Teachers completed a web-based questionnaire taking 15–20 min and were compensated with a gift certificate worth 100 SEK. Altogether, the combined teacher–student data covers 169 lower secondary school units, with a response rate of 78% for grade 9 students to the SSS (*n* = 10,757), and 54.3% for teachers to the STS (*n* = 2304).

**Fig. 2:**
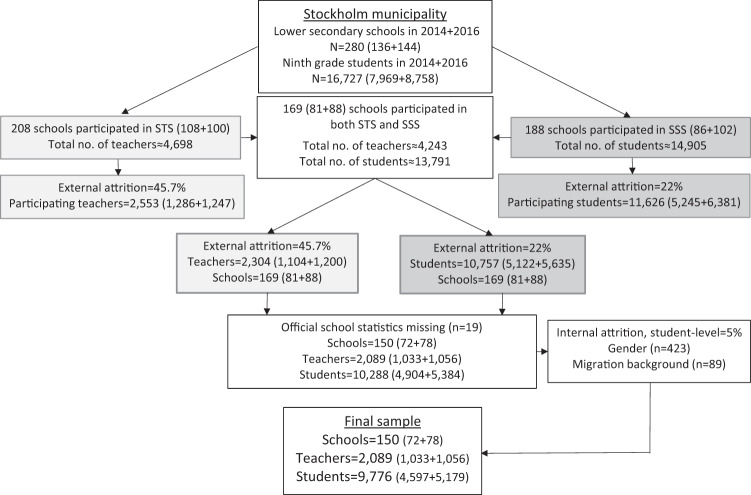
Overview of the process whereby the final numbers of schools, teachers and students in the data were obtained, with the total population of ninth-grade students in Stockholm municipality in 2014 and 2016 as the point of departure

Teacher reports on school ethos were aggregated to school-level means and then merged with individual student-level data information, enabling the analysis of school-level variables and student-reported outcomes. Complete information on all the variables used in the analyses was available for 9776 ninth-grade students (*n* = 4597 in 2014; *n* = 5179 in 2016) distributed over 150 school units (*n* = 72 in 2014; *n* *=* 78 in 2016). Aggregated information on school ethos for these 150 school units were based on a total of 2089 teacher ratings (*n* = 1033 in 2014; *n* = 1056 in 2016). Figure 2 presents an overview of the participations rates, with the total population of ninth-grade students in Stockholm municipality in 2014 and 2016 as the point of departure.

Data from the 2014 and 2016 surveys were used to maximise statistical power. Thus, participating grade 9 students from each year were unique observations. While some teachers are likely to have been represented in both years of the survey, the school context assessed by teachers was nonetheless distinctive for each school year due to changes in the student body composition, the school’s leadership as well as staff turnover.

### Measures

#### School ethos

School ethos captured teacher reports of school values and norms. It is a unidimensional school-level composite index that was developed in a previous study (Granvik Saminathen et al. 2018). Teachers responded to 12 items about perceptions of the school’s clarity of values, teachers’ motivation and expectations of students, the strength of interventions against bullying and violence, student–teacher relationships, as well as the rate of staff turnover. Each item had the response alternatives: “Strongly agree”, “Agree”, “Neither agree nor disagree”, “Disagree”, and “Strongly disagree” (Kjellström et al. 2017). Teachers’ responses were summed to form an index that was then aggregated to the school’s mean response. See Table 1 for descriptive statistics and model fit.

**Table 1:** Descriptive statistics and model fit of school ethos (*n* = 2351 teachers, *n* = 176 lower secondary school units)

Teacher-rated school ethos	Loadings	RMSEA	TLI	CFI	Mean (SD)	Range	Cronbach’s Alpha
CFA 1 factor model fit		0.09	0.92	0.93		15–59	0.90
At this school we have a value system (värdegrund) which is clear to students	0.70				2.18 (0.90)	1–5	
At this school the teachers make an effort to provide positive feedback about students’ performance	0.76				1.85 (0.71)	1–5	
Teachers have high expectations of student performance	0.77				1.84 (0.76)	1–5	
Teachers take their time with students even if they want to discuss something other than school work	0.73				1.87 (0.77)	1–5	
At this school we actively work on issues such as violence, bullying and harassment among students	0.72				1.98 (0.89)	1–5	
This school provides a stimulating learning environment	0.64				2.56 (0.96)	1–5	
The teachers at this school have a strong work ethic	0.80				1.80 (0.79)	1–5	
The teachers work with strong enthusiasm	0.83				2.11 (0.85)	1–5	
At this school the students are treated with respect	0.82				1.69 (0.71)	1–5	
The teachers at this school feel confident as classroom leaders	0.80				2.08 (0.82)	1–5	
At this school students’ motivation is a stimulating part of work	0.68				2.34 (0.99)	1–5	
There is high staff turnover amongst teachers at this school	0.32				2.75 (0.92)	1–4	

#### Psychological distress

Psychological distress represented students’ internalising signs of poor psychological well-being. Three items assessed the frequency of students’ depressive feelings (“Do you feel sad and depressed without knowing why?”), anxious feelings (“Do you ever feel frightened without knowing why?”) and “enjoyment of life” (“How often do you feel it is really good to be alive?”). The response options included: “Seldom”, “Occasionally”, “Sometimes”, “Pretty often”, and “Very often”, with a higher score indicating higher levels of psychological distress. The final item was reverse coded.

#### Aggressive behaviour

Aggressive behaviour addressed externalising signs of poor psychological well-being using the following four statements: “I do the opposite of what people tell me to do just to make them angry”, “I can’t stand being provoked—it makes me want to hit someone”, “If I get angry with someone, I don’t think twice about hurting him/her”, and “I’ll attack anyone who makes me angry—even though he/she didn’t hit me first”. The response options were: “Describes very poorly”, “Describes rather poorly”, “Describes rather well”, and “Describes very well”, with a higher score indicating higher levels of aggressive behaviour.

#### Academic achievement

Academic achievement consisted of the three school performance indicators available in the SSS, namely individual student-reported marks from the previous term in the core subjects Swedish, English and mathematics. Response options for each subject were “No mark recorded” (student did not receive a mark in this subject) (=0); “Fail” (=0); “E” (=1); “D” (=2); “C” (=3); “B” (=4); and “A” (=5).

Four control variables were included in the model to account for students’ own sociodemographic background and the student intake at the school.

#### Parental education

Parental education was measured by the question “Which is the highest education of your parents?” (mother and father separately). Four response options were provided: “Comprehensive school”, “Secondary school”, “University and University College”, and “I don’t know”. The responses were recoded into a 3-category variable representing “No parent with university education *or* information missing”, “One parent with university education” and “Two parents with university education”.

#### Migration background

The question “How long have you lived in Sweden?” served as a proxy for *migration background* with the response alternatives “All my life”, “10 years or more”, “5–9 years” and “Less than 5 years”. Responses were recoded to “No migration background”, “Lived in Sweden 10 years or more” and “Lived in Sweden 9 years or less”. These categories (approximately) differentiate between children of immigrants who arrived in Sweden before starting compulsory school and those who arrived in Sweden after having reached the school starting age. *Family structure* was also included, distinguishing between students who reported living with both parents and those who did not. Further, the analyses adjusted for individual student *gender*.

#### School student body composition

A four-category measure of sociodemographic school segregation was used to control for school student body composition, reflecting “Privileged” (C1), “Typical” (C2), “Deprived” (C3) and “Deprived immigrant schools” (C4). This measure was developed in a previous publication (Granvik Saminathen et al. 2018) that used latent class analysis to identify four clusters of schools with similar student composition profiles according to the following criteria: parents’ average education, proportion of students born abroad, proportion of recently immigrated students (i.e., within the past 4 years), and students’ average academic motivation. Information for the first three criteria was provided by the Swedish National Agency of Education (SNAE), and data for the fourth criterion was derived from students’ own ratings in the SSS, aggregated to the school level.

Descriptive statistics of the student variables as well as the distribution of students over the four school composition profiles are presented in Table 2.

**Table 2:** Descriptive statistics (*n* = 9776 students, *n* = 150 schools)

Dependent variables	Mean	SD	Range
Psychological distress (latent)			
Feeling sad	2.6	1.3	1–5
Feeling anxious	1.6	1.0	1–5
(Not) loving life	2.2	1.2	1–5
Aggressive behaviour (latent)			
Hurt	2.0	1.1	1–4
Attack	1.5	0.8	1–4
Provoke	1.9	0.9	1–4
Go against	1.7	0.8	1–4
Academic achievement (latent)			
Swedish	2.8	1.3	0–5
English	3.2	1.4	0–5
Mathematics	2.7	1.5	0–5

Control variables	*N*	%
Gender		
Boys	4918	50.3
Girls	4858	49.7
Parent(s) with university education		
None or information missing^a^	4130	42.3
One	2135	21.8
Two	3511	35.9
Migration background		
No	8189	83.8
In Sweden 10 years or more	720	7.3
In Sweden 9 years or less	867	8.9
Student composition profiles		
Privileged schools	1757	18.0
Typical schools	5482	56.1
Deprived schools	1113	11.4
Deprived immigrant schools	1424	14.5

^a^Includes *n* = 2896 with information missing

### Statistical Analyses

All analyses were conducted in Mplus Version 8.4 (Muthén and Muthén 2017). Due to the hierarchical clustering of students within schools, multilevel structural equation modelling (ML-SEM) was applied, with students defined at Level 1 (L1) and teachers (i.e., schools) at Level 2 (L2). Following a two-step model-building process, the measurement model and then the structural model was tested. Multilevel SEM takes within-group dependency into account and correctly estimates standard errors (Heck and Thomas 2015), while modelling latent factors and simultaneously testing direct and indirect effects (Preacher et al. 2010). The maximum likelihood estimator MLR was used, which is robust to non-normality and non-independence of observations (Maydeu-Olivares 2017), and full information maximum likelihood estimation (FIML) was used to handle missing data. Among the analysed students, 9.1% had missing data on at least one item. The chi-square test statistic, the Comparative Fit Index (CFI), the Tucker–Lewis index (TLI), and the Root Mean Square Error of Approximation (RMSEA) were used to assess model fit. According to Marsh et al. (2004), CFI and TLI values above 0.90 indicate acceptable fit, while values above 0.95 point to a good fit (Marsh et al. 2004). Consistent with Hu and Bentler (1999), RMSEA values below 0.08 and 0.06 were considered as indicative of acceptable and excellent model fit, respectively.

First, intra-class correlations (ICC) for academic achievement, psychological distress and aggressive behaviour were assessed, determining the proportion of variance related to between-school differences. Next, the measurement model was examined through multilevel Confirmatory Factor Analysis (ML-CFA) to confirm the factor structure of the measurement model (Brown 2015) and equality of the factor loadings across the within and between levels. The factorial invariance of the measurement model across gender and year was examined by performing multigroup CFA. Finally, the structural model was estimated using ML-SEM. Such analyses evaluate if the factor structure of the three latent variables fit in a similar way and if scale items function similarly across different groups (Meade and Lautenschlager 2004). If model fit does not decrease significantly as equality constraints across groups are imposed on features of the model, measurement invariance is confirmed (Byrne et al. 1989; Steenkamp and Baumgartner 1998). Partial invariance can be confirmed if the parameters of at least two indicators per construct are equal across groups in terms of loadings and intercepts (Pendergast et al. 2017). Typically, chi-square difference tests indicate invariance in a model, but in large samples chi-square tends to be oversensitive and thus nearly always large and statistically significant (Chen 2007; Dumenci and Achenbach 2008). Consequently, CFI was evaluated, as this model fit statistic is less sensitive to model complexity and sample size, following Cheung and Rensvold’s (2002) recommendations for invariances tests (∆CFI ≤ 0.01).

## Results

### Sample Descriptives

Descriptive statistics of the student variables as well as the distribution of students over the four school composition profiles are provided in Table 2. Girls represented 49.7% of the study sample. The majority of the participating students (83.8%) have lived in Sweden all of their lives, and 57.7% reported that they have at least one parent with a university education. More than half (56.1%) of the participants attended schools that are labelled as *typical*. The schools belonging to this cluster are the most sociodemographically heterogeneous, with levels of parental education, foreign-born and recently immigrated students falling somewhere in between the privileged and the two deprived segregation profiles.

### Measurement Model

The ICCs revealed that 23.2% of the variance in academic achievement was explained at the school-level. For psychological distress and aggressive behaviour, a lower proportion of variance was attributable to the school level, 2.4% and 6.4%, respectively. Thus, differences between schools were greater for aggressive behaviour than for psychological distress.

The suitability of the measurement model was examined through a ML-CFA, which showed acceptable model fit for the three-factor measurement model: *X*^2^ = 800.63; *df* = 64, *p* < 0.001; CFI = 0.962; TLI = 0.947; RMSEA = 0.033 (see Table 3). At the individual level, all factor loadings were satisfactory, with standardised loadings ranging between 0.47 and 0.88, except for the observed variable “If I get angry with someone, I don’t think twice about hurting him/her” (*hurt*). This item had a relatively low factor loading (0.35) on the latent variable *aggressive behaviour*. As this item is theoretically a good fit and does not necessarily compromise the validity of the measurement model, the item was retained. At the school-level, factor loadings were also satisfactory, with standardised loadings ranging between 0.70 to 1.00. Constraining the factor loadings across the student and school levels did not reduce the model fit, supporting the invariance of the factor loadings across the levels (see Table 3). Correlations among the latent factors are presented in Table 4. Measurement invariance was observed across survey years (2014 and 2016) and partial measurement invariance was observed across gender (see Table 3). The intercepts for items *loving life* and *Swedish* were non-invariant, indicating that at a given level of the latent construct (*psychological distress* and *academic achievement*, respectively) boys were more likely than girls to endorse higher categories of *loving life* and *Swedish*.

**Table 3:** 3-Factor measurement model fit and measurement invariance

Model	*X*^2^	*df*	CFI	TLI	RMSEA	Test for invariance (compared to less constrained model)	
						∆X^2^	∆CFI
ML-CFA							
No constraints across levels	800.63	64	0.962	0.947	0.033		
Factor loadings constrained	900.01	74	0.957	0.949	0.032	*p* < 0.001	0.005
Survey year							
2014	381.16	32	0.963	0.948	0.046		
2016	332.17	32	0.973	0.962	0.041		
Configural	716.13	64	0.969	0.956	0.044		
Metric invariance	694.91	71	0.970	0.962	0.041	*p* > 0.05	0.001
Scalar invariance	655.80	78	0.972	0.968	0.037	*p* < 0.05	0.002
Gender							
Boys	175.07	32	0.983	0.976	0.029		
Girl**s**	287.62	32	0.976	0.967	0.039		
Configural	460.83	64	0.979	0.971	0.035		
Metric invariance	550.38	71	0.975	0.968	0.036	*p* < 0.001	0.004
Scalar invariance	1310.92	78	0.935	0.925	0.056	*p* < 0.001	>0.01
Partial scalar invariance	643.09	75	0.970	0.964	0.038	*p* < 0.001	0.005

∆*X*^*2*^ = uses the Satorra–Bentler scaling correction; Configural invariance = no constraints across groups; Metric invariance = factor loadings constrained; Scalar invariance = factor loadings + intercepts constrained; Partial scalar invariance = factor loadings + intercepts constrained except for *(not) loving life* and *Swedish*

**Table 4:** Correlations among the latent factors at the student- and school levels

	Psychological distress		Aggressive behaviour	
	Student level	School level	Student level	School level
Aggressive behaviour	0.144***	0.371		
Academic achievement	−0.022	−0.424	−0.331***	−0.789

***p < 0.001

### Predicting Academic Achievement and Psychological Well-Being

**Fig. 3:**
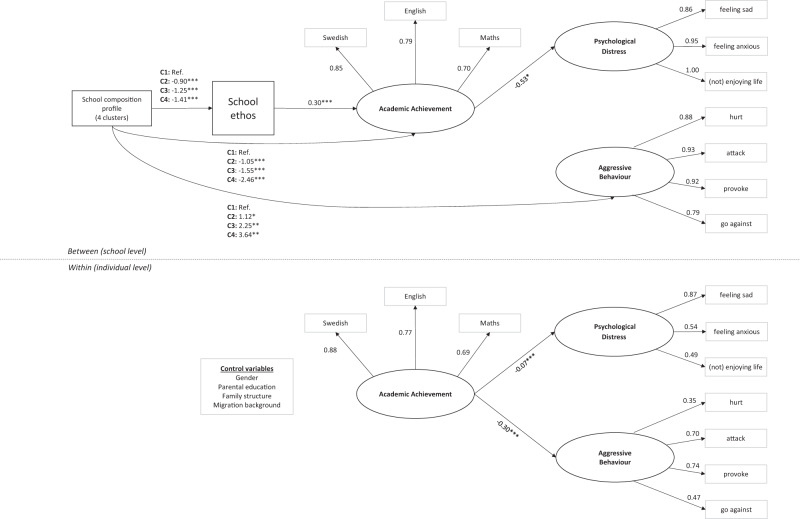
Final model (*n* = 9776 students). C1 = Privileged, C2 Typical, C3 = Deprived and C4 = Deprived immigrant schools

The structural model was then evaluated using ML-SEM. The student-level control variables were included as predictors of each of the three latent factors. As no significant association between school composition profile and psychological distress could be identified, this path was removed. The final model fit the data reasonably well (*X*^2^ = 1956.39; *df* = 138, *p* < 0.001; CFI = 0.924; TLI = 0.894; RMSEA = 0.037). See Fig. 3 for results for the final model. Measurement invariance for the school-level factor school ethos across years was confirmed (data not shown).

Firstly, as hypothesised, a school’s student body composition was significantly associated with the school’s average academic achievement and level of aggressive behaviour among students, to the disadvantage of more sociodemographically deprived schools. The school’s student body composition significantly predicted teacher-rated school ethos, with less privileged schools presenting with lower levels of school ethos, on average. As expected, stronger ratings of school ethos were significantly related to higher average academic achievement among students, beyond the effects of the student- and school-level sociodemographic control variables. Continuing at the school-level, no significant direct associations between school ethos and psychological distress or aggressive behaviour were identified. However, higher average academic achievement among students was significantly associated with lower psychological distress in the student population. Achievement mediated the effect of school ethos on psychological distress, indicating an indirect pathway for school ethos (b = −0.003, *p* = 0.023). However, the relationship between academic achievement and aggressive behaviour at the school-level was not statistically significant. At the student-level, higher academic achievement in core subjects was significantly associated with lower levels of both psychological distress and aggressive behaviour (Fig. 3).

Finally, to test whether the effects of school ethos on student outcomes differed between boys and girls, cross-level interactions between school ethos and gender for the three student outcomes were examined. No significant interactions were observed, indicating that the associations between school ethos and each of *academic achievement* (b = 0.006, *p* *=* 0.337), *psychological distress* (b = 0.000, *p* *=* 0.988) and *aggressive behaviour* (b = 0.000, *p* *=* 0.868) did not vary between boys and girls. No significant gender moderation effects were observed at the student-level either, indicating that academic achievement was associated with boys’ and girls’ psychological distress and aggressive behaviour to a similar extent.

## Discussion

A high-quality educational and social school environment can promote adolescents’ school performance but also their concurrent and future psychological well-being (Ahrén 2010; PHAS 2018a, 2018b). As school-contextual characteristics are potentially alterable through effective management and constructive educational policies, it is critical to identify links between effective school features and student outcomes. School ethos defined as a measure of a school’s purposeful efforts to develop the learning environment and relationships between staff and students has the potential to promote both students’ academic achievement (Jacobson 2011) and reduce poor psychological well-being (Aldridge and McChesney 2018). Thus, strengthening the school’s ethos could particularly benefit more sociodemographically disadvantaged schools attempting to compensate for students’ family background. Through multilevel structural equation modeling, the current study examined the role of teacher-rated school ethos in relation to students’ academic achievement as well as internalising and externalising manifestations of poor psychological well-being, taking school student body composition into account.

In line with the study’s first hypothesis, the association between school ethos and the school’s level of academic achievement indicates that it is possible for socioeconomically disadvantaged schools to compensate for students’ family background by systematically implementing and enforcing policies that strengthen the shared values and normative practices at the school, in line with the effective schools theories. In fact, some of the more sociodemographically “deprived” schools in this study present with rather strong teacher-ratings of school ethos (results not shown) (Granvik Saminathen et al. 2018). Thus, this study advocates that by cultivating positive common beliefs, values and norms at a school, it may be possible to promote positive academic outcomes, regardless of the students’ family background and the school’s sociodemographic composition.

Although the direct paths between school ethos and psychological distress and aggressive behaviour were not significant, school ethos indirectly predicted psychological distress through academic achievement. Thus, although the school’s primary task is teaching and preparing students for future studies and employment, this study suggests that a school’s ethos may also be indirectly relevant for students’ level of psychological well-being, at least in terms of psychological distress. The findings of this study are particularly pertinent in light of a recent publication by the Swedish Public Health Agency (PHAS 2018a) that indicated an association between shortcomings in the Swedish school system and the increase in multiple health complaints among adolescents. The conclusions of this PHAS report acknowledge the importance of schools and school quality for adolescent psychological well-being. In Sweden, grade nine is the last year of compulsory schooling and students tend to be aware of the competition surrounding the admission to upper secondary school, particularly the most prestigious ones, placing great emphasis on school performance (Giota and Gustafsson 2017). The high pressure to perform to secure future educational and employment opportunities or the distress of receiving poor academic outcomes during this school year could thus have negative effects on students’ psychological well-being. Creating a school environment that supports students in coping with these school demands is thus imperative.

Further, the study found that there were no significant gender differences in the association between school ethos and academic achievement or between achievement and students’ psychological well-being. This indicates that the pathway between school ethos and academic achievement and in turn the association with indicators of poor psychological well-being is similar for boys and girls.

In view of rising school segregation in Sweden, the findings regarding the school’s sociodemographic composition are also worth highlighting. Firstly, the significant association between a school’s sociodemographic composition and school ethos was anticipated, based on previous findings (Granvik Saminathen et al. 2018; Clotfelter et al. 2005). In line with an earlier study (Granvik Saminathen et al. 2018), a school’s sociodemographic profile was not only directly associated with student achievement, but the effect of the school’s student body composition on academic achievement also seemed to be operating indirectly through school ethos. Thus, while schools with motivated students and few socioeconomically disadvantaged children may have more advantageous preconditions for creating a stable and stimulating learning atmosphere (Palardy 2008), more socially disadvantaged schools could improve student performance through determined organisational efforts to build a high-quality learning environment (Hopson and Lee 2011). This could allow schools located in the most socially disadvantaged areas to deal with challenges associated with the unequal distribution of students across schools.

By combining survey data with official data on school student composition, this study was able to examine the role of school-contextual features for adolescent outcomes, as well as the implications of a segregated school landscape. The multilevel analytical approach further strengthened the study design by avoiding downwardly biased standard errors in the results. Using multi-informant data with survey information from both teachers and students minimised bias related to common method variance (Podsakoff et al. 2003). Teachers’ assessment of the school’s normative practices is expected to be more objective, as students’ perceptions of the school’s ethos may depend on their school performance and/or overall well-being. Finally, by considering both internalising and externalising problems, the study has highlighted that despite common gender differences in expressions of poor psychological well-being, the strength of associations with school ethos and academic achievement were similar for boys and girls.

Nonetheless, the cross-sectional design limits interpretations about causality. Bearing in mind the mutual relationship between academic achievement and adolescent psychological well-being, a cross-sectional study is unable to exclude the possibility of reverse or reciprocal causation. However, although some students may achieve low academic results because of preceding poor psychological well-being, robustness analyses (results not shown) that tested an alternative causal ordering, with psychological well-being predicting academic achievement, yielded no significant results, strengthening the current model. In order to come closer to causal pathways between school performance and well-being, longitudinal studies would be valuable.

In addition, as the analyses are based on data from Stockholm municipality, the results may be specific to the distinctive features and challenges of the Swedish education system (Organisation for Economic Co-operation and Development 2016), as well as the comparatively high prevalence of poor psychological health among adolescents in Sweden (Bremberg 2015). Nevertheless, it is likely that similar associations between school ethos, academic achievement and poor psychological health may be observed among schools in other Swedish municipalities as well as in other countries.

Future research may also want to investigate the effects of school ethos on particular groups of students, for example high-achieving girls, who tend to report poor psychological well-being despite comparatively high achievement (Låftman et al. 2013). It could also be relevant to focus on children from more disadvantaged family backgrounds, as they may be particularly likely to benefit from a school that offers the characteristics ascribed to a positive social school environment (Hopson and Lee 2011). The extent to which the effects of school ethos and achievement hold for different degrees of psychological ill health could also be investigated, employing scales that capture more or less severe symptoms.

In pursuit of deepening the knowledge about the mechanisms behind the associations between school ethos and student outcomes, future research should examine which specific indicators of school ethos are most relevant to students’ outcomes. After all, it is highly demanding and resource-intensive to improve features such as teacher support and the school learning environment (Bragg and Manchester 2011).

## Conclusion

Considering the school’s compensatory duty, equal access to a high-quality school context is critical for adolescents’ future opportunities and well-being. In order to inform efforts of schools to make a difference for their students regardless of the school’s sociodemographic student composition, research on contextual features of schools in relation to student academic and health-related outcomes is vital. The current study contributed to this literature by examining academic achievement as a potential mechanism linking school ethos to school-level psychological distress and aggressive behaviour. The results demonstrated that teacher-rated school ethos was significantly associated with a school’s average marks in core subjects, highlighting the importance for school management to facilitate the building of a strong and sustainable ethos in lower secondary schools. Furthermore, schools with higher average academic achievement were significantly more likely to have students with lower average levels of psychological distress at the school. At the individual level, students with higher academic achievement tended to report both less psychological distress and aggressive behaviour, confirming the relationship between adolescents’ academic achievement and psychological well-being. Overall, these findings suggest that when teachers rate a school’s ethos as positive, students at the school perform better academically, on average, regardless of their own family background. Higher school performance, in turn, is associated with fewer emotional problems among students.
